# Multisystem Sarcoidosis Presenting as Severe Hypercalcemic Crisis and Generalized Lymphadenopathy Mimicking Hematological Malignancy: A Case Report

**DOI:** 10.7759/cureus.112447

**Published:** 2026-07-11

**Authors:** Aye Nyein Thu, Munir Babar, Yamna Seneen, Payal Subhash Joshi

**Affiliations:** 1 Diabetes and Endocrinology, Alexandra Hospital, Worcestershire Acute Hospitals NHS Trust, Redditch, GBR; 2 Geriatrics, Alexandra Hospital, Worcestershire Acute Hospitals NHS Trust, Redditch, GBR; 3 General Medicine, Alexandra Hospital, Worcestershire Acute Hospitals NHS Trust, Redditch, GBR

**Keywords:** generalized lymphadenopathy, haematological malignancy, hypercalcemia, hypercalcemic crisis, lymphoma, multisystem sarcoidosis, sarcoidosis, sarcoidosis hypercalcemia

## Abstract

Sarcoidosis is a multisystem granulomatous disorder of unknown etiology characterized histologically by the formation of non-caseating granulomas in various organs and tissues. Typically, sarcoidosis is recognized by features such as dry cough, fever, erythema nodosum, and bilateral hilar lymphadenopathy. However, when patients present with severe hypercalcemia and generalized lymphadenopathy, sarcoidosis is usually overlooked, and we usually investigate for features of malignancy, bone metastasis, lymphoma, multiple myeloma, and primary hyperparathyroidism. We report the case of a 61-year-old lady who presented with severe hypercalcemia and generalized lymphadenopathy; further investigations ultimately led to the diagnosis of sarcoidosis with multisystem involvement. This case illustrates the importance of considering a broader differential diagnosis in diagnostically challenging cases, especially when typical etiologies are not identified, as early diagnosis and prompt treatment are essential. It also highlights the importance of involving a multidisciplinary team early in the diagnostically challenging cases with multisystem involvement when initial investigations fail to identify more common causes.

## Introduction

Sarcoidosis is a multisystem granulomatous disorder of unknown etiology characterized by the presence of non-caseating granulomas in various organs throughout the body [1,2]. The disease demonstrates marked geographical variation, with the highest incidence rates reported in northern Europe and colder regions of the world [2]. Sarcoidosis is usually known as a benign, self-limiting disease with a good prognosis [2]. Acute manifestations of sarcoidosis, especially Löfgren syndrome, which is characterized by fever, erythema nodosum, migratory polyarthritis, and bilateral hilar lymphadenopathy (BHL), are readily recognized by clinicians [1,2]. However, presentations dominated by severe hypercalcemia and generalized lymphadenopathy are relatively less common in sarcoidosis and can be life-threatening [1-3], and usually they lead clinicians to the suspicion of malignancy. Additionally, peripheral generalized lymphadenopathy is less common than hilar or mediastinal lymphadenopathy [1-3]. Furthermore, when generalized lymphadenopathy co-occurs with systemic constitutional symptoms, suspicion is naturally directed toward hematological malignancies, particularly lymphoma.

Severe hypercalcemia represents a critical medical emergency that may lead to cardiac arrhythmias and sudden death [2]. Early diagnosis and treatment are essential as diagnostic uncertainties can lead to delayed intervention and life-threatening complications. Corticosteroids are the first-line treatment for sarcoidosis, whereas as methotrexate, hydroxychloroquine, azathioprine, leflunomide, and mycophenolate mofetil can be added as steroid-sparing agents or second-line treatments in resistant cases [1,2]. Third-line options include anti-tumor necrosis factor (TNF) inhibitors such as infliximab or adalimumab. Dose and duration of steroids depend on the type of organ involvement and severity [1]. However, recent evidence from the Prednisone versus Methotrexate (PREDMETH) trial demonstrated that methotrexate was non-inferior to prednisone as first-line treatment for improving lung function at 24 weeks in patients with pulmonary sarcoidosis [4]. Chronic sarcoidosis with multisystem involvement requires careful monitoring and regular follow-up before being discharged back to primary care [1,2]. Early involvement of a multidisciplinary team is crucial in those complicated cases with diagnostic challenges to rectify the definitive diagnosis and prompt treatment [2].

We report a case of a 61-year-old woman presenting with severe hypercalcemia, weight loss, and widespread lymphadenopathy mimicking an advanced hematological malignancy, who was ultimately diagnosed with active multisystem sarcoidosis. The patient initially received intravenous fluids and zoledronic acid for management of hypercalcemia, followed by oral corticosteroid therapy, resulting in normalization of serum calcium levels and clinical improvement.

This case highlights an uncommon presentation of multisystem sarcoidosis with severe hypercalcemia and generalized lymphadenopathy closely mimicking hematological malignancy. Clinicians should consider sarcoidosis in the differential diagnosis of unexplained hypercalcemia and widespread lymphadenopathy after exclusion of more common causes. Early multidisciplinary assessment and tissue diagnosis are crucial to avoid delays in definitive treatment.

## Case presentation

History and physical examination

A 61-year-old woman presented to the accident and emergency (A&E) department experiencing generalised malaise, severe fatigue, myalgia, exertional dyspnea, and an unintentional weight loss of approximately one stone (14 lbs) over the preceding month. Over the week prior to admission, her symptoms escalated to include severe nausea, loss of appetite, mild abdominal pain, and recurrent vomiting. She denied experiencing fever, night sweats, cough, recent viral illnesses, or changes in bowel habits. Her medical history was notable only for fibromyalgia; however, she emphasized that her current symptoms were entirely distinct from her typical fibromyalgia flares. She was a non-smoker, rarely consumed alcohol, and explicitly denied taking calcium, vitamin D, or over-the-counter multivitamin supplements.

On physical examination, the patient was alert and oriented with a Glasgow Coma Scale (GCS) of 15/15 but appeared visibly unwell and dehydrated. Her body mass index (BMI) was 28.3 kg/m², and she did not appear cachectic. Broad clinical palpation revealed no obvious superficial lymphadenopathy in the cervical or axillary chains, and no hepatosplenomegaly was initially felt. There was no spinal or bony tenderness, and no purpura, bruising, or lower-limb skin lesions (such as erythema nodosum) were present. Vital signs were stable with a regular pulse and a blood pressure of 111/71 mmHg. Respiratory and cardiovascular auscultations were unremarkable.

Diagnostic workup

Initial laboratory investigations (Table 1) revealed severe hypercalcemia, with an adjusted serum calcium level of 3.96 mmol/L.

**Table 1:** Initial laboratory investigations eGFR: estimated glomerular filtration ratio; ALKP: alkaline phosphatase; ALT: alanine aminotransferase; GGT: gamma glutamyl transferase.

Parameter	Baseline patient value	Reference range	Clinical interpretation
Haemoglobin (Hb)	127 g/L	115-164 g/L	Normal
White Blood Cell Count (WBC)	7.6 x 10^9^/L	4.0-11.0 x 10^9^/L	Normal
Platelet Count (Plt)	260 x 10^9^/L	150 – 400 x 10^9^/L	Normal
C-Reactive Protein (CRP)	24 mg/L	<5 mg/L	Insignificantly elevated
eGFR	61 mL/min	>60 mL/min	Normal
Creatinine	88 µmol/L	49-90 µmol/L	Normal
Sodium (Na)	143 mmol/L	133-146 mmol/L	Normal
Potassium (K)	3.5 mmol/L	3.5-5.3 mmol/L	Normal
Adjusted calcium	3.96 mmol/L	2.2-2.6 mmol/L	Significantly elevated
Albumin	41 g/L	35-50 g/L	Normal
Phosphate	0.87 mmol/L	0.80-1.50 mmol/L	Normal
ALKP	408 IU/L	30-130 IU/L	Significantly elevated
ALT	41 IU/L	0-35 IU/L	Slightly elevated
GGT	853 IU/L	<38 IU/L	Significantly elevated
Bilirubin	11 µmol/L	5-21 µmol/L	Normal
Parathyroid Hormone (PTH)	0.8 pmol/L	1.3-9.3 pmol/L	Suppressed
Vitamin D	48 nmol/L	>50 nmol/L	Mild insufficiency

Accompanying this was a marked cholestatic liver enzyme derangement, characterized by significantly elevated gamma-glutamyl transpeptidase (GGT) and alkaline phosphatase (ALKP) levels. Full blood count, renal function tests, and serum albumin were within normal reference ranges. Importantly, parathyroid hormone (PTH) levels were profoundly suppressed (0.8 pmol/L), ruling out primary hyperparathyroidism, while a serum vitamin D level of 48 nmol/L excluded vitamin D intoxication. 

**Figure 1:**
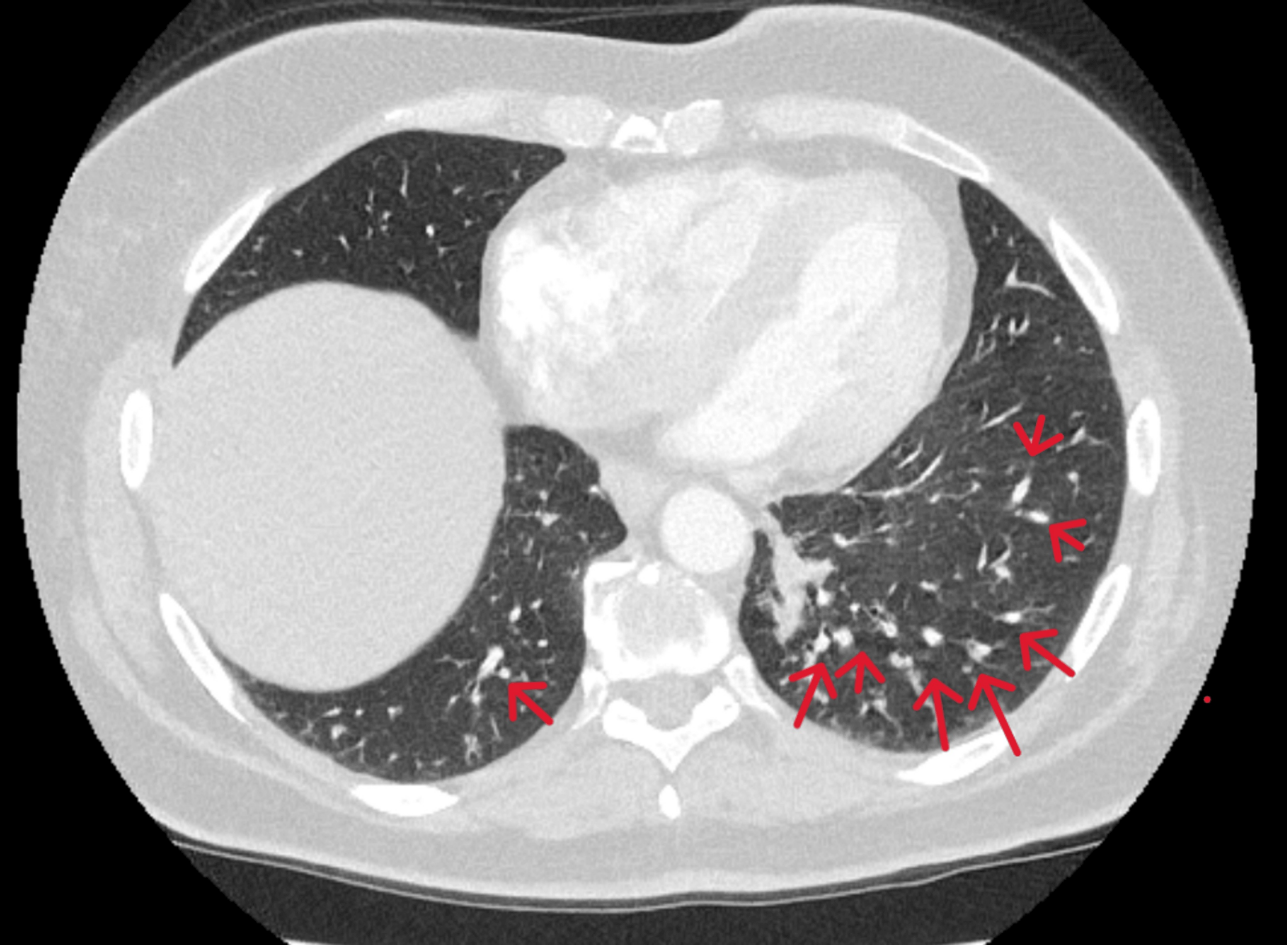
CT chest showing scattered tiny nodules in both lungs

**Figure 2:**
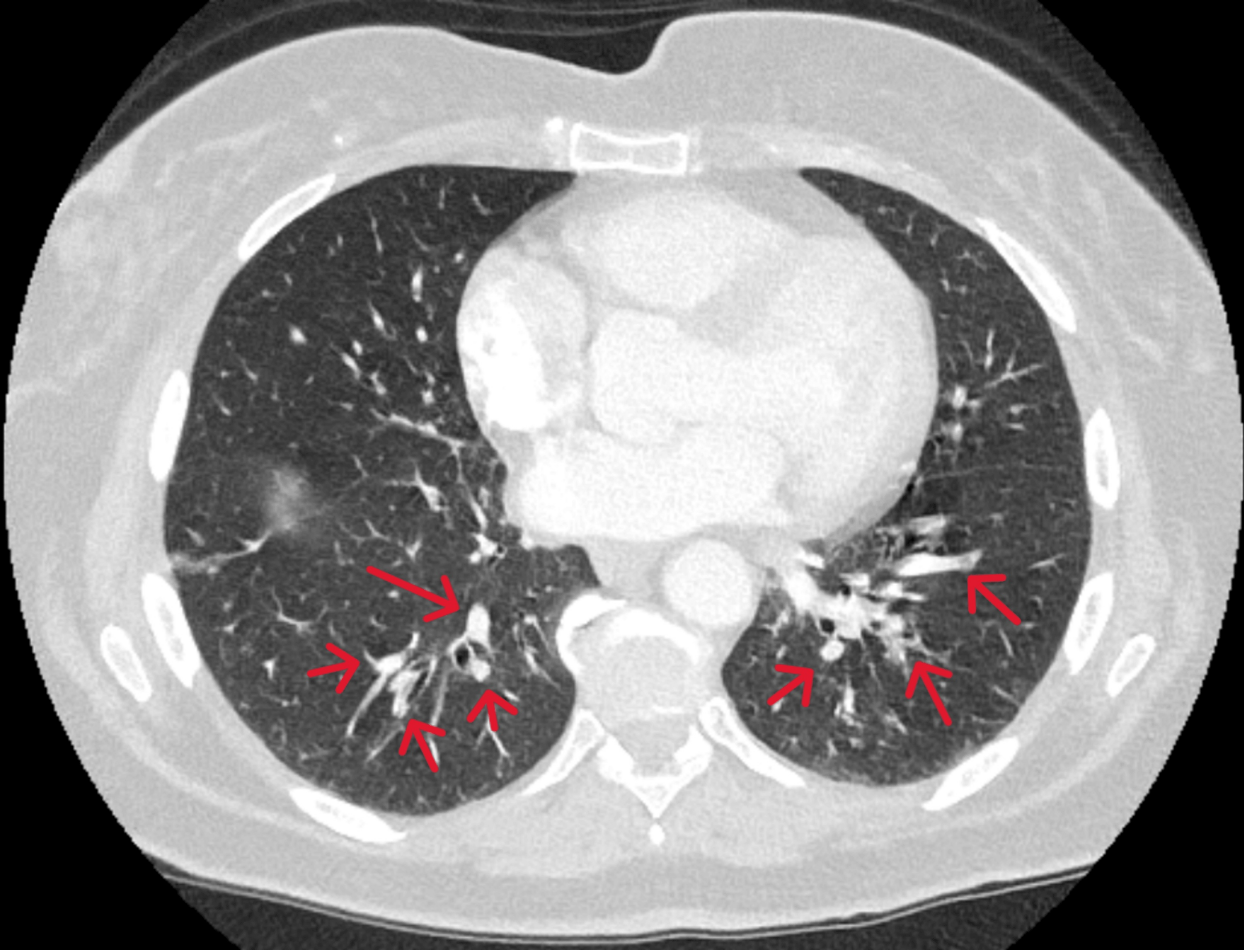
CT chest showing thin soft tissue along the bronchovascular bundle in the hilar region Scattered tiny nodules in both lungs along with branching irregular opacity in the left lower lobe posterior basal segment.

**Figure 3:**
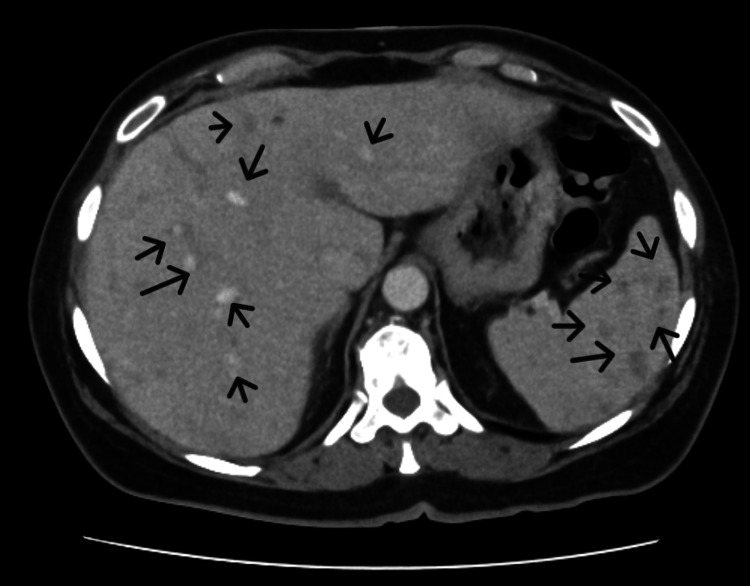
CT abdomen and pelvis revealed enlarged liver, heterogenous parenchyma with ill-defined patchy low density lesions Enlarged spleen with several small focal lesions.

**Figure 4:**
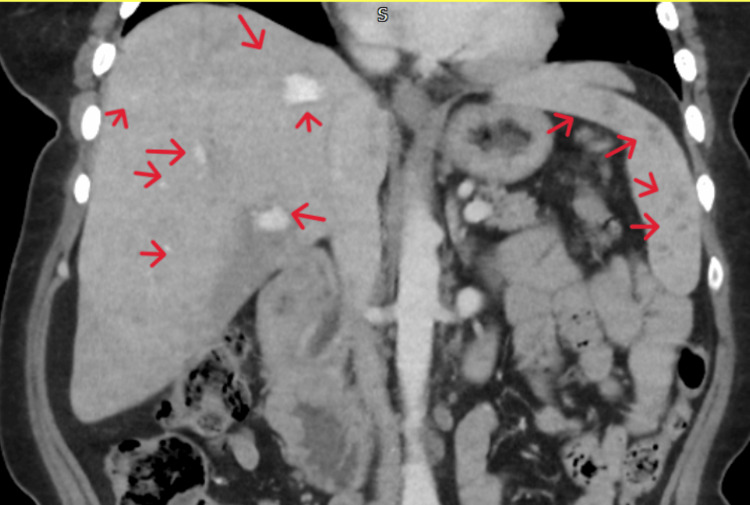
CT abdomen and pelvis showing hepatomegaly with multiple focal lesions in the liver and spleen, no abdominal or pelvic lymphadenopathy

Given the severe hypercalcaemia, suppressed PTH, and significant weight loss, a computed tomography (CT) scan of the thorax, abdomen, and pelvis (TAP) (Figures* *1-4) was emergently performed to evaluate for occult malignancy or widespread metastases.

The CT scan revealed a complex clinical picture: extensive bilateral cervical and axillary lymphadenopathy, multiple scattered pulmonary nodules, hepatosplenomegaly with discrete focal hypodense lesions in both the liver and spleen, and indeterminate lucent bone lesions. The findings of multiple lesions in the liver (Figures 3, 4) with liver enzyme derangement (Table 1) were consistent with hepatic involvement by sarcoidosis, where non-caseating granulomatous inflammation and granuloma formation within the liver can cause intrahepatic cholestasis and impaired bile flow.

The case was urgently reviewed by the hematology team due to high suspicion of a hematological malignancy, such as lymphoma or multiple myeloma. An intensive diagnostic workup was initiated (Table* *2), including serum protein electrophoresis, serum free light chains, lactate dehydrogenase (LDH), and viral serologies, all of which yielded unremarkable or negative results.

**Table 2:** Secondary diagnostic and malignancy screening workup IgA: Immunoglobulin A; IgG: Immunoglobulin G; Ig M: Immunoglobulin M; CMV: Cytomegalovirus; EBV: Epstein-Barr Virus.

Parameter	Result/Value	Reference range	Clinical interpretation
Plasma protein electrophoresis	Raised a1+2 and b2 regions	No discrete bands	Unremarkable; no M-protein band
IgA	3.4 g/L	0.7-4.0 g/L	Normal
IgG	11.3 g/L	7-16 g/L	Normal
IgM	1.2 g/L	0.4-2.3 g/L	Normal
Kappa free light chains	43.16 mg/L	3.3-19.4 mg/L	Elevated
Lambda free light chains	40.63 mg/L	5.7-26.3 mg/L	Elevated
Kappa/Lambda ratio	1.062	0.260-1.650	Normal ratio; excludes myeloma
Lactate dehydrogenase (LDH)	163 U/L	120-250 U/L	Normal
Hepatitis B & C serology	Not detected	Negative	Normal
Hepatitis A serology	IgG positive	Negative	Consistent with past infection
CMV & EBV serology	Negative	Negative	Normal

To obtain a definitive tissue diagnosis, the respiratory multidisciplinary team (MDT) was consulted for a potential bronchoscopy or endobronchial ultrasound (EBUS)-guided lung biopsy. The MDT concluded that the pulmonary nodules did not present an accessible or safe target for EBUS/bronchoscopy and advised an image-guided peripheral lymph node biopsy instead. Consequently, the patient underwent an ultrasound-guided core biopsy of a right axillary lymph node under interventional radiology guidance.

Histopathological analysis of the lymphoid core (Table 3) revealed multiple well-formed, non-caseating (non-necrotizing) granulomas with no evidence of malignant cells.

**Table 3:** Histopathology and specific biomarker assessment ACE: Angiotensin-converting enzyme.

Parameter/Procedure	Microscopy and histology findings	Clinical interpretation
Right axillary lymph node biopsy	Core showing multiple sarcoid-like non-caseating granulomas; no malignant cells isolated.	Non-necrotizing granulomatous lymphadenitis consistent with sarcoidosis
Ziehl–Neelsen (ZN) stain	Negative	Mycobacterium tuberculosis highly unlikely
Periodic Acid–Schiff (PASD) stain	Negative	Fungal etiology excluded
Serum ACE levels	82 U/L (Reference: <52 U/L)	Elevated; supports active sarcoidosis

Special staining using Ziehl-Neelsen (ZN) for acid-fast bacilli and Periodic Acid-Schiff-Diastase (PASD) for fungal structures were both negative, effectively ruling out tuberculosis and deep fungal infections. Additionally, serum angiotensin-converting enzyme (ACE) levels were found to be elevated at 82 U/L (Table 3), further supporting the diagnosis of active, systemic sarcoidosis. The case was concurrently reviewed by the oncology MDT, which agreed that the extensive visceral nodularities and hepatosplenomegaly were entirely secondary to active multisystem sarcoidosis rather than metastatic disease.

Management and clinical course

The patient's initial hypercalcemic crisis was treated aggressively with intensive intravenous fluid resuscitation and a single infusion of intravenous zoledronic acid (4 mg). Her serum calcium levels gradually fell over several days following this intervention (Table 4).

**Table 4:** Longitudinal adjusted serum calcium levels

Clinical context and therapy status	Adjusted calcium level (mmol/L)	Comment
On admission	3.96	Severe hypercalcemic crisis
2 days post-intravenous bisphosphonate & fluid resuscitation	3.52	Reducing trend
On discharge	2.98	Showing a downward trend
Outpatient review; completely normalized on prednisolone 20 mg OD	2.51	Normalized
Clinical/biochemical relapse following a taper to prednisolone 10 mg OD	2.77	Relapsed
Normalized following dose escalation back to prednisolone 20 mg OD	2.56	Normalized again

Once histopathological confirmation of sarcoidosis was achieved, she was formally discharged into the care of the respiratory outpatient clinic for systemic management. She was commenced on oral prednisolone 20 mg once daily for one month, to be followed by a planned maintenance dose of 10 mg once daily for six to 12 months. Co-prescriptions included alendronic acid (70 mg once weekly) and omeprazole (10 mg once daily) for bone and gastrointestinal prophylaxis during prolonged steroid use.

During a subsequent taper down to 10 mg once daily, the patient suffered a biochemical and symptomatic relapse (Table 4), with her adjusted calcium rising to 2.77 mmol/L. The respiratory team promptly restored the prednisolone dose back to 20 mg once daily, which successfully re-established and maintained normocalcemia. 

## Discussion

Hypercalcemia occurs in approximately 5-10% of patients diagnosed with sarcoidosis, but a presentation featuring a severe, life-threatening hypercalcemic crisis is exceedingly rare [3,5]. The physiological driver behind this metabolic abnormality involves the autonomous hyperproduction of calcitriol (1,25-dihydroxyvitamin D) by extrarenal 1a-hydroxylase, an enzyme highly expressed by the activated macrophages residing within the sarcoid granulomas [2,3]. This hyperproduction leads to uncontrolled intestinal calcium absorption and bone resorption, independent of parathyroid hormone control. Consequently, sarcoidosis represents an essential diagnostic consideration in any patient presenting with PTH-independent hypercalcemia, especially when malignancy has been ruled out [3,5].

Acute sarcoidosis is usually considered a benign, self-limiting disease in most cases [1,2]. Most people with sarcoidosis continue to live a normal life [1,2]. BHL and stage I radiographic changes (hilar and mediastinal lymphadenopathy alone) in the lungs usually resolve [1,2]. However, multisystem involvement with an insidious form of chronic progressive sarcoidosis requires a multidisciplinary team approach and monitoring [1,2]. Factors influencing the prognosis of sarcoidosis include age over 40 years and major organ involvement, including central nervous system (CNS), renal (nephrocalcinosis), and pulmonary involvement with stage III (pulmonary infiltrates alone) and IV (pulmonary fibrosis) radiographic changes [1]. Patients with a chronic form of disease require initiation of steroid therapy and monitoring [1]. The duration of therapy depends on the response to treatment and resolution of symptoms as well as biochemical and radiological changes [1]. Although life-threatening manifestations of sarcoidosis are relatively uncommon, severe hypercalcemic crises represent a significant clinical challenge and can be life-threatening [6-9].

The coexistence of generalized lymphadenopathy, prominent constitutional symptoms, multiple pulmonary nodules, and focal lesions throughout the liver and spleen strongly mimicked lymphoma or disseminated metastatic malignancy [6,8]. Such overlapping clinical presentations routinely trigger exhaustive, invasive oncological investigations before a benign granulomatous disease is even considered. Histopathological confirmation remains the absolute diagnostic gold standard [2,10]. It requires the definitive demonstration of non-caseating granulomas alongside careful special staining (such as ZN and PASD) to completely exclude infectious mimics like tuberculosis or fungal infections [2,10].

This challenging case underscores the value of an early, well-coordinated multidisciplinary approach. Collaboration across acute medicine, haematology, respiratory medicine, oncology, interventional radiology, and pathology prevented diagnostic delay and facilitated rapid tissue acquisition. Corticosteroids remain the absolute first-line therapeutic standard for active sarcoidosis with severe hypercalcaemia or extensive multisystem involvement [1,10]. In cases resistant to steroids, second-line disease-modifying anti-rheumatic drugs (DMARDs) like methotrexate, azathioprine, or leflunomide are utilized, while anti-TNF agents (e.g., infliximab) serve as third-line options [1,11].

The primary educational takeaway from this case is that sarcoidosis can perfectly mirror advanced disseminated malignancies. Clinicians must maintain a broad differential diagnosis when managing unexplained hypercalcemia and widespread lymphadenopathy to achieve an accurate diagnosis and implement timely treatment.

## Conclusions

Multisystem sarcoidosis can present atypically as a severe hypercalcemic crisis paired with widespread lymphadenopathy, closely mimicking advanced lymphomas or metastatic disease. When classic etiologies like primary hyperparathyroidism or malignancy are excluded, sarcoidosis must be strongly considered within the differential diagnosis. Granulomatous disease like sarcoidosis should be considered in PTH-independent hypercalcemia with multisystem involvement. Systemic corticosteroids are highly effective in managing granuloma-mediated hypercalcemia, though long-term monitoring is essential due to the high risk of relapse during steroid tapering. Ultimately, a multidisciplinary approach is vital for achieving rapid tissue diagnosis and initiating prompt, life-saving therapeutic interventions.
